# Down-Regulation of microRNA-26a Promotes Mouse Hepatocyte Proliferation during Liver Regeneration

**DOI:** 10.1371/journal.pone.0033577

**Published:** 2012-04-04

**Authors:** Jian Zhou, Weiqiang Ju, Dongping Wang, Linwei Wu, Xiaofeng Zhu, Zhiyong Guo, Xiaoshun He

**Affiliations:** Organ Transplant Center, the First Affiliated Hospital, Sun Yat-sen University, Guangzhou, China; National Cancer Institute, United States of America

## Abstract

**Background:**

Inadequate liver regeneration (LR) is still an unsolved problem in major liver resection and small-for-size syndrome post-living donor liver transplantation. A number of microRNAs have been shown to play important roles in cell proliferation. Herein, we investigated the role of miR-26a as a pivotal regulator of hepatocyte proliferation in LR.

**Methodology/Principal Findings:**

Adult male C57BL/6J mice, undergoing 70% partial hepatectomy (PH), were treated with Ad5-anti-miR-26a-LUC or Ad5-miR-26a-LUC or Ad5-LUC vector via portal vein. The animals were subjected to *in vivo* bioluminescence imaging. Serum and liver samples were collected to test liver function, calculate liver-to-body weight ratio (LBWR), document hepatocyte proliferation (Ki-67 staining), and investigate potential targeted gene expression of miR-26a by quantitative real-time PCR and Western blot. The miR-26a level declined during LR after 70% PH. Down-regulation of miR-26a by anti-miR-26a expression led to enhanced proliferation of hepatocytes, and both LBWR and hepatocyte proliferation (Ki-67^+^ cells %) showed an increased tendency, while liver damage, indicated by aspartate aminotransferase (AST), alanine aminotransferase (ALT) and total bilirubin (T-Bil), was reduced. Furthermore, CCND2 and CCNE2, as possible targeted genes of miR-26a, were up-regulated. In addition, miR-26a over-expression showed converse results.

**Conclusions/Significance:**

MiR-26a plays crucial role in regulating the proliferative phase of LR, probably by repressing expressions of cell cycle proteins CCND2 and CCNE2. The current study reveals a novel miRNA-mediated regulation pattern during the proliferative phase of LR.

## Introduction

After 70% partial hepatectomy (PH) in mice, the residual liver is unique in its intrinsic ability to regenerate to restore its original mass and function within 7–10 days in a process called liver regeneration (LR) [1]–[6]. Despite there are massive studies of LR, many aspects of this process remain still unknown, for example, the elegant genetic regulation of hepatocytes proliferation.

MicroRNAs (miRNAs) are a class of small RNAs regulating gene expression by degrading messenger RNAs via binding to their 3′-untranslated regions (3′-UTRs). MiRNAs have been reported to modulate a variety of biological processes, including cell differentiation, proliferation, metabolism, apoptosis and even carcinogenesis [7]–[11]. Several studies have shown the critical role of miRNAs in liver regeneration. Song GS et al reported that miR-378 plays critical roles during the early phase of LR by directly inhibiting the expression of *Odc1*, which is associated with DNA synthesis [12]–[13]. Furthermore, miR-26a has been reported to be involved in various cell functions [14]–[16]. Kota J et al have shown that miR-26a presented with an anti-proliferative property in human liver cancer [15], and another study also showed that miR-26a family members suppressed tumorigenesis in B lymphoma cells [17]. These reports promote us to investigate the role of miR-26a in hepatocyte proliferation during LR. In our preliminary study, using a quantitative real-time PCR analysis, we found that, like miR-378, miR-26a expression was obviously down-regulated in regenerating mice liver tissue at 120 h after 70% PH, compared with the sham operation (SH) group. We therefore hypothesized that down-regulation of miR-26a might promote hepatocyte proliferation during LR.

In the current study, we showed evidences that miR-26a expression is remarkably declined during LR after PH, and that down-regulation of this miRNA could promote hepatocyte proliferation *in vivo*. MiR-26a may regulate LR by repression of cell cycle proteins CCND2 and CCNE2. This study provides a novel mechanism and potential therapeutic target of miRNA regulation of hepatocyte proliferation during LR.

## Materials and Methods

### Animals

A total of 120 healthy male C57BL/6J mice (purchased from the Animal Center of Sun Yat-sen University, Guangzhou, China), aged 8–10 weeks and weighing 21–25 g, were housed (2–4 mice per cage) in an animal room under specific pathogen-free conditions with 22±2.0°C indoor temperature and a 12-hour light/dark cycle, and had free access to water and standard chow. All animal experiments were performed in a humane manner, and in accordance with the Institutional Animal Care Instructions. The study protocol was approved by the Ethics Committee for Animal, Sun Yat-sen University (approval ID: 2010 NO.9).

### Vector Construction

Firstly, LUC and IRES were cloned into a pShuttle-CMV vector (Agilent Technologies, USA), and then pri-miR-26a sequences or anti-miR-26a sequences were introduced into the pShuttle-CMV-IRES-LUC vector (Agilent Technologies, USA). The pShuttle-CMV-IRES-LUC vector after linearization with PmeI and pAdWasy-1 (Agilent Technologies, USA) was recombined into pAdEasy-IRES-LUC vector. Next, a 293AD cell line (Cell Biolabs, San Diego, USA) [18], was transfected with pAdEasy-IRES-LUC vector, and then, liquid supernatant including viral particles was isolated and collected. The viral particles including Ad5-miR-26a-LUC vector, Ad5-anti-miR-26a-LUC vector, were established.

### Transfection Efficiency Assessment

The Ad5-miR-26a-LUC vector and Ad5-anti-miR-26a-LUC vector were diluted to different concentrations of 4×10^10^ IU/mL, 4×10^8^ IU/mL and 4×10^6^ IU/mL with PBS, respectively. Each vector was transfected to mice. Three days later, the liver tissue was collected to test the expression of miR-26a by real time PCR.

### Surgical Procedure

Forty animals were randomly divided into two groups (n = 20 in each group) as follows: (1) In PH group, 70% PH was performed under anesthesia with isoflurane as described by Mitchell *et al* [4]. In brief, the left lateral, median liver lobes were surgically removed after laparotomy. (2) In SH group, the abdomen of mice was opened but no liver resection was performed. In the functional study, 80 mice undergoing 70% PH, were randomly assigned to four groups (n = 20 in each group) as follows: (1) In Ad5-anti-miR-26a-LUC (AA) group, animals were treated with Ad5-anti-miR-26a-LUC vector (0.5 mL, 4×10^10^ IU/mL) via portal vein. (2) In Ad5-miR-26a-LUC (AM) group, animals were treated with Ad5-miR-26a-LUC vector (0.5 mL, 4×10^10^ IU/mL) via portal vein. (3) In Ad5-LUC (AL) group, animals were treated with Ad5-LUC empty vector (0.5 mL, 4×10^10^ IU/mL) via portal vein. (4) In control group, animals only received 70% PH but with no transfection. At the indicated time points (24 h, 72 h, 120 h, 168 h after resection), the mice were sacrificed and the residual liver specimens and blood samples were collected for analysis.

### *In vivo* Bioluminescence Imaging

At 24h or 72 h after transfection, mice from the AA group (n = 5), AM group (n = 5), AL group (n = 5) and control group (n = 5), were subjected to *in vivo* bioluminescence imaging [19]–[24]. Briefly, the animals, anaesthetized by isoflurane as described previously [4], were intraperitoneally injected with D-luciferin (Biotium, USA) in a concentration of 150 mg/Kg, and 20 minutes later, were subjected to the *in vivo* bioluminescence imaging using the system of photobiology (Zhongke, China).

### Liver-to-body Weight Ratio

At the indicated time points, the animals were sacrificed. The total body weight was measured and the remnant and regenerated liver tissues were resected and weighed. The acquired data were expressed as percentage of the ratio between remnant liver weight (A), divided by the total body weight (B) times 100 (liver-to-body weight ratio [LBWR] (%) = A/B×100).

### Liver Function Tests

Mice were sacrificed and blood samples were collected via the postorbital venous plexus. Blood serum was sampled and analyzed for aspartate aminotransferase (AST), alanine aminotransferase (ALT), and total bilirubin (T-Bil) using methods as described [25].

### Immunohistochemical Staining and Evaluation

Mice liver tissues were collected at the indicated time points from the AA group, AM group, AL group and control group. Immunostaining for Ki-67, a marker for cell proliferation, was performed to evaluate the proliferation of hepatocytes according to the manufacture’s guidelines. The primary antibody was a rabbit monoclonal anti-mouse/rat/human Ki-67 antigen (DCS Diagnostics, Germany). Immunohistochemistry was performed using a biotin-free enhanced polymer one-step staining technique (EPOS-method) with a peroxidase-conjugated polymer backbone coupled with a goat anti-rabbit secondary antibody (Dako, Germany). “Proliferation index” was defined as the percentage of Ki-67 positive cells randomly counted in five high-power fields (×400) of each specimen.

### Quantitative Real-time PCR (qRT-PCR)

Total RNA was extracted from prepared liver samples with Trizol (Invitrogen, Carlsbad, USA) reagent and cDNA was synthesized according to the manufacturer’s protocol (MBI Fermentas). Quantitative RT-PCR was performed using a standard SYBR Green PCR Master Mix (Toyobo, Osaka, Japan), and PCR-specific amplification was conducted in the Applied Biosystems (ABI7500) real-time PCR machine. The relative expression of genes (miR-26a, U6, CCND2, CCNE2, CCNE1, CDK6, CCND1, CCND3, and β-actin) was calculated with the 2-(ΔΔCt) method [26]. The primers used are listed in Table 1.

**Table 1:** Primers used in reverse transcription and quantitative real-time PCR.

miRNA and genes	Primers sequences
miR-26a forward	5'-ACACTCCAGCTGGGTTCAAGTAATCCAGGATAGGC
miR-26a reverse	5'-CTCAACTGGTGTCGTGGA
U6 forward	5'-CTCGCTTCGGCAGCACA
U6 reverse	5'-AACGCTTCACGAATTTGCGT
CCND2 forward	5'-CCAGACTGTGCCTTGGGAAT
CCND2 reverse	5'-GACACAGGGACAAGTGTGGT
CCNE2 forward	5'-CTGCTGCCGCCTTATGTCAT
CCNE2 reverse	5'-TACACACTGGTGACAGCTGC
CCNE1 forward	5'-GTTACAGATGGCGCTTGCTC
CCNE1 reverse	5'-ACCCGTGTCGTTGACATAGG
CDK6 forward	5'-TAGCTGTCTCCACCACCCAC
CDK6 reverse	5'-GGCCATCTGTCGTTAGCCAG
CCND1 forward	5'-GGATGCTGGAGGTCTGTGAG
CCND1 reverse	5'-CTTAGAGGCCACGAACATGC
CCND3 forward	5'-GAATGATGGCAGTGGATGGA
CCND3 reverse	5'-GCACGCACTGGAAGTAGGAG
β-actin forward	5'-CGCCACCAGTTCGCCATGGATGA
β-actin reverse	5'-CCACATAGGAGTCCTTCT

### Western Blot Analysis

For whole protein extracts, liver tissue samples after grinding were homogenized in lysis buffer (Promega, USA), incubated for 30 minutes on ice, then centrifuged for 15 min at 14000×g. Prior to use, all buffers were treated with a protease inhibitor cocktail (Konchem, China). Equal amounts of protein were separated discontinuously on 12–15% SDS-PAGE and transferred to a PVDF membrane (Millipore, USA). The antibodies employed included anti-CCND2 (Santa cruz, USA), anti-CCNE2 (Santa cruz, USA), anti-CCNE1 (Santa cruz, USA), anti-CDK6 (Santa cruz, USA), anti-CCND1 (Santa cruz, USA), anti-CCND3 (Santa cruz, USA) and β-actin (Kangcheng, China). Immunoblots were developed using anti-mouse- or anti-rabbit-HRP secondary antibodies (Dako, CA), followed by detection with immobilon western chemilimunescent HRP substrate (Millipore, USA) according to the manufacturer’s instructions. For all western blots, β-actin was used as a reference gene.

### Statistical Analysis

All data are expressed as mean ± standard deviation. The statistical analysis was performed by one-way analysis of variance. A *P* value of less than 0.05 was considered to be statistically significant.

## Results

### Down-regulated miR-26a Expression During LR

**Figure 1:**
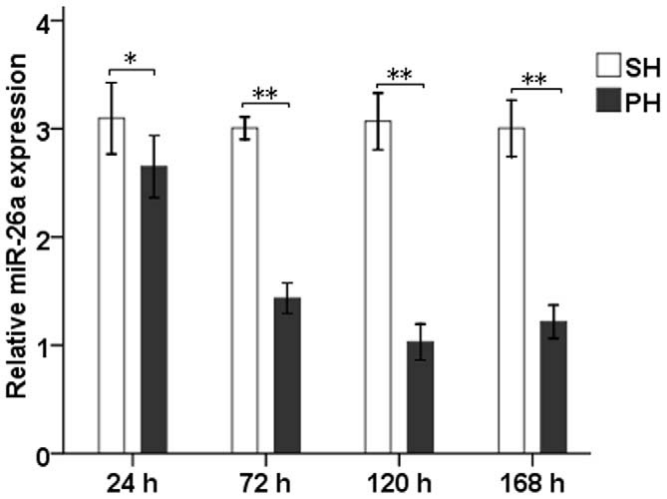
**Down-regulation of miR-26a during LR.** The expression of miR-26a in the regenerating liver from 24 h to 168 h after 70% PH was assessed by qRT-PCR analysis. MiR-26a levels were standardized to that of U6. All data were obtained from at least three independent experiments and are shown as the means ± S.D., ^*^ *P*<0.05, ^**^ *P*<0.01.

We measured the mRNA expression of miR-26a during LR using qRT-PCR at indicated time points. The miR-26a levels declined during LR after 70% PH, as observed in the regenerating mouse liver compared with SH group. This difference was most significant at 120 h after PH, when the miR-26a expression showed a 3-fold reduction compared to SH group (*P*<0.01) (Figure 1).

### Transfection Efficiency and Analysis of *in Vivo* Bioluminescence Imaging

**Figure 2:**
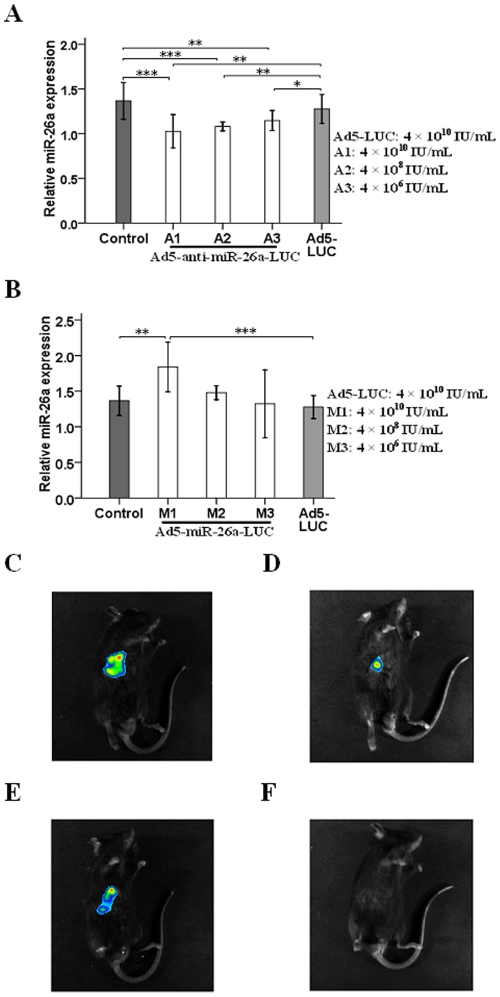
**Transfection efficiency and** ***in vivo*** **bioluminescence imaging.** (A) Expression of miR-26a after transfection with different concentrations of Ad5-anti-miR-26a-LUC, Ad5-LUC, or no transfection (control). (B) Expression of miR-26a after transfection with different concentrations of Ad5-miR-26a-LUC, Ad5-LUC, or no transfection (control). The degree of bioluminescence was the greatest in Ad5-anti-miR-26a-LUC (AA) group (C), less in Ad5-LUC (AL) group (E), and the weakest in Ad5-miR-26a-LUC (AM) group (D). The mice in control group showed no bioluminescence image (F). ^*^ *P*<0.05, ^**^ *P*<0.01, ^***^ *P*<0.001.

To assess transfection efficiency of vector *in vivo*, we measured miR-26a expression in mice transfected with Ad5-anti-miR-26a-LUC, Ad5-miR-26a-LUC or Ad5-LUC vector. The miR-26a expressions after transfection with Ad5-anti-miR-26a-LUC in concentrations of 4×10^10^ IU/mL (A1), 4×10^8^ IU/mL (A2) and 4×10^6^ IU/mL (A3) were decreased compared with control group (1.02±0.07 vs. 1.36±0.08, *P*<0.001; 1.08±0.02 vs. 1.36±0.08, *P*<0.001; 1.14±0.04 vs. 1.36±0.08, *P*<0.01). Similar results in A1, A2 and A3 group were observed compared with Ad5-LUC group (1.02±0.07 vs. 1.27±0.06, *P*<0.01; 1.08±0.02 vs. 1.27±0.06, *P*<0.01; 1.14±0.04 vs. 1.27±0.06, *P*<0.05) (Figure 2A). In contrast, increased miR-26a expression after transfection with Ad5-miR-26a-LUC in concentration of 4×10^10^ IU/mL (M1) was observed compared with control group (1.84±0.14 vs. 1.36±0.08, *P*<0.01). Likely, significant difference can also be seen between sub-group M1 and Ad5-LUC group (1.84±0.14 vs. 1.27±0.06, *P*<0.001) (Figure 2B). In addition, there is no significant difference between Ad5-LUC group and control group. Besides, the *in vivo* bioluminescence imaging technology was used to verify that Ad5-anti-miR-26a-LUC (AA), Ad5-miR-26a-LUC (AM) and Ad5-LUC (AL) have been successfully transfected into the liver of C57BL/6J mice. At 24 h after transfection, the area of bioluminescence in each group showed no obvious difference (Fig. S1). And at 72 h after transfection, because of the different size of regenerated liver masses, the area of bioluminescence was obviously different (Figure 2C, D, E), and the mice in control group showed no bioluminescence (Figure 2F).

### Impacts of miR-26a Transfection on LBWR in C57BL/6J Mice after 70% PH

**Figure 3:**
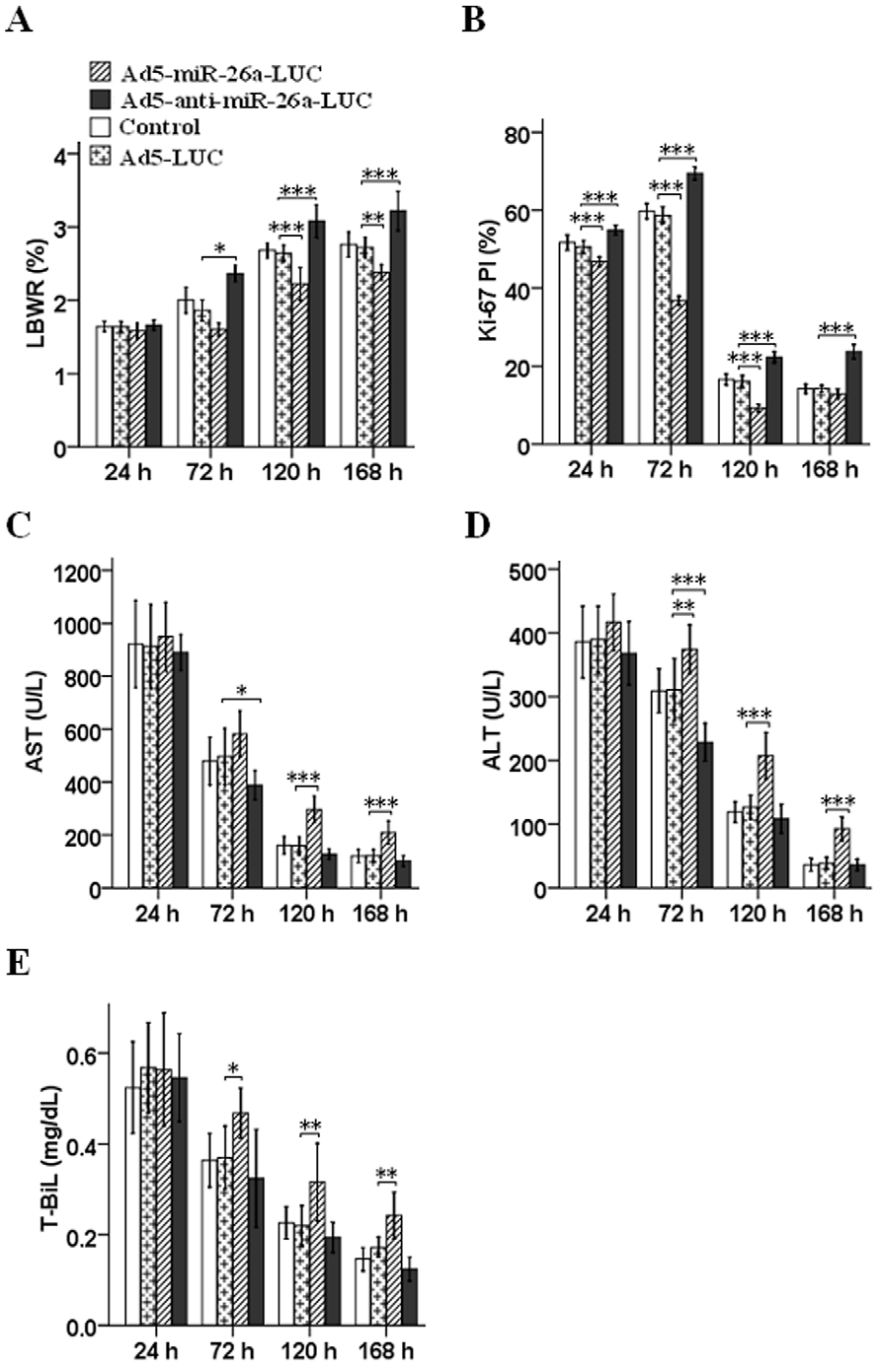
**Anti-miR-26a expression promotes liver regeneration and improves liver function in mice.** (A) LBWR of mice transfected with Ad5-anti-miR-26a-LUC (AA), Ad5-miR-26a-LUC (AM) and Ad5-LUC (AL). There was an increased LBWR in AA group compared to AL group (*P*<0.001), and a decreased LBWR in AM group can be seen compared with AL group at 120 h (*P*<0.001). (B) The Ki-67 proliferation index (PI) after 70% PH and transfection, was significantly higher in AA group compared with AL group (*P*<0.001), while lower in AM group in comparison with AL group (*P*<0.001). (C-E) Liver function tests after transfection, worse liver functions could be observed in AM group compared with AL group. ^*^ *P*<0.05, ^**^ *P*<0.01, ^***^ *P*<0.001.

To investigate the impacts of miR-26a on regulating LR after 70% PH in C57BL/6J mice, we assessed the LBWR after 70% PH. We observed a higher LBWR (2.22±0.35%) in AA group compared with AL group (1.86±0.11%) at 72 h after transfection (*P*<0.05). Likely, at 120 h and 168 h after transfection, higher LBWRs were also observed in AA than AL group (3.08±0.17% vs. 2.64±0.08%, *P*<0.001; 3.22±0.21% vs. 2.72±0.1%, *P*<0.001), and reverse results were obtained between AM group and AL group (120 h: 2.22±0.17% vs. 2.64±0.08%, *P*<0.001; 168 h: 2.38±0.08% vs. 2.72±0.1%, *P*<0.01). The LBWR of control group were 1.64±0.05%, 2.00±0.14%, 2.68±0.08% and 2.76±0.13%, respectively, at 24 h, 72 h, 120 h and 168 h after 70% PH. In addition, there were no statistical differences between control group and AL group at all time points. The LBWRs in different groups were shown in Figure 3A.

### Impact of miR-26a Transfection on Hepatocyte Proliferation after 70% PH

As expected, the increase of liver mass was accompanied by a corresponding increase of the Ki-67 proliferation index (PI). After transfection, a significant increase of the Ki-67 PI was seen in AA group when compared with AL group (24 h: 54.90±0.98% vs. 50.60±1.26%, *P*<0.001; 72 h: 69.48±1.35% vs. 58.72±1.71%, *P*<0.001; 120 h: 22.26±1.09% vs. 16.16±1.18%, *P*<0.001; and 168 h: 23.68±1.49% vs. 14.3±0.63%, *P*<0.001). In contrast, a significant decrease was observed in AM group in comparison with AL group (24 h: 46.82±0.95% vs. 50.60±1.26%, *P*<0.001; 72 h: 36.80±0.94% vs. 58.72±1.71%, *P*<0.001; and 120 h: 9.30±0.75% vs. 16.16±1.18%, *P*<0.001). The Ki-67 PI of control group was 51.70±1.58%, 59.78±1.55%, 16.60±1.11% and 14.22±0.93%, respectively, at 24 h, 72 h, 120 h and 168 h after 70% PH. In addition, there was no statistical difference between control group and AL group at all time points. The Ki-67 PIs in different groups were shown in Figure 3B.

### Impact of miR-26a Transfection on Liver Function Tests after 70% PH

To assess liver cell injury after transfection, we accessed plasma liver function including AST, ALT and T-Bil. More serious liver damage was documented in AM group than in AL group (shown in Figure 3C, D, E). In addition, there was no statistical difference between control group and AL group at all time points.

### CCND2 and CCNE2 but not CCNE1, CDK6, CCND1 or CCND3 are Potential Targeted Genes of miR-26a

**Figure 4:**
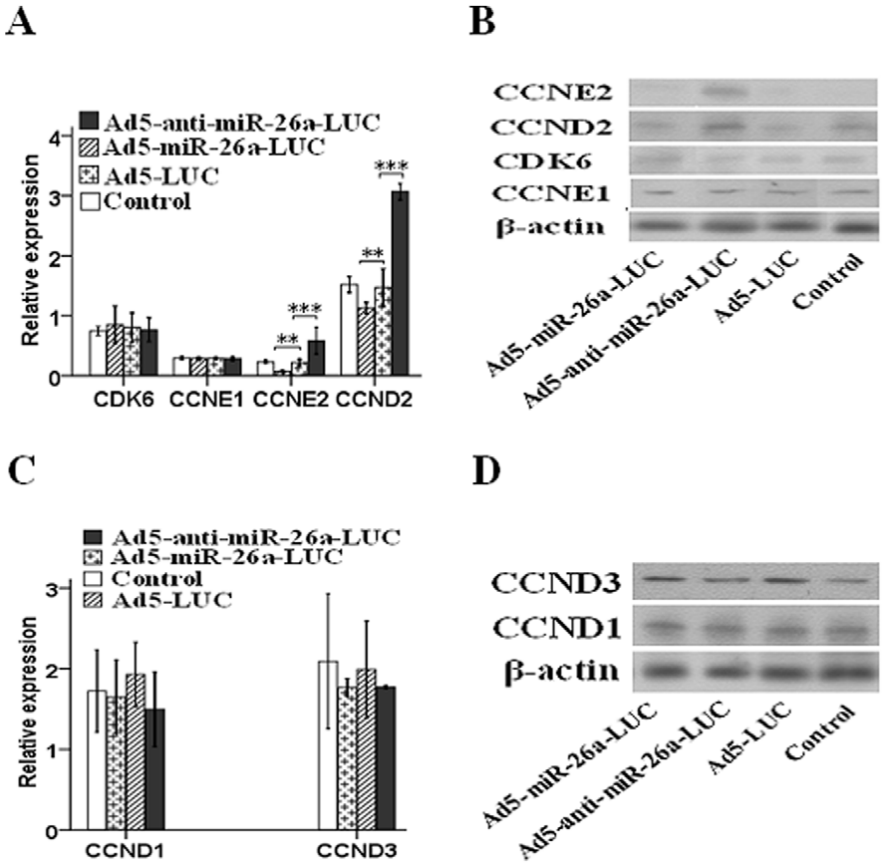
**CCND2 and CCNE2 are potential targeted genes of miR-26a.** (A) Anti-miR-26a expression increased the mRNA expression of CCND2 and CCNE2 as shown by qRT-PCR. Conversely, miR-26a over-expression declined the mRNA expression of the two genes. The mRNA expression of CCNE1 and CDK6 showed no obvious change. (B) Anti-miR-26a expression up-regulated the protein expression of CCND2 and CCNE2. In contrast, miR-26a over-expression down-regulated the protein expression of CCND2 and CCNE2. The protein expression of CCNE1 and CDK6 showed no obvious change. (C and D) Expression of CCND1 and CCND3 in both mRNA and protein level showed no obvious changes. ^*^ *P*<0.05, ^**^ *P*<0.01, ^***^ *P*<0.001.

To investigate the mechanism through which miR-26a modulates the cell cycle of proliferative phase of liver cells, we examined putative targets of miR-26a using algorithms including Targetscan, miRanda, and PicTar [27]–[29]. These analysis predict that miR-26a may regulate cyclin D2 (CCND2), cyclin E1 (CCNE1), cyclin E2 (CCNE2), and cyclin dependent kinase 6 (CDK6), all of which play an important role in cell cycle [30]. Quantitative real-time PCR and western blotting were used to determine whether miR-26a regulates any of these putative targets *in vivo*. Both CCND2 and CCNE2 expression in AA group were notably enhanced in both mRNA (Figure 4A) and protein level (Figure 4B). In contrast, they were remarkably decreased in AM group. However, the CCNE1 and CDK6 expression showed no apparent change in both mRNA (Figure 4A) and protein level (Figure 4B). The CCND1 and CCND3 expression in AA group and AM group showed no obvious change in both mRNA (Figure 4C) and protein level (Figure 4D) compared with AL group. In addition, there is no significant difference between control group and AL group. Furthermore, the CCND2, CCNE2, CDK6 and CCNE1 expression *in vitro* in both mRNA and protein level also supported the *in vivo* findings (Fig. S2). Together, these results suggest that CCND2 and CCNE2 but not CCNE1, CDK6, CCND1 or CCND3 are potential targeted genes of miR-26a.

## Discussion

Loss of liver mass triggers a regenerative response in the whole liver. Liver tissue loss may occur as a result of partial liver resection, living donor liver transplantation, reduced size liver transplantation, toxic injury, exposure to viruses and trauma. If LR fails to give a prompt and sufficient response to the loss, the patients would die off liver failure [31]. Although there are evidences that several miRNAs are involved in LR [12], [32]–[34], little is known about how these miRNAs regulate the proliferative phase of LR. Recent studies showed that miR-26a is down-regulated in breast cancer and nasopharyngeal carcinoma [35], [36], indicating that it is a pivotal miRNA regulating cell proliferation. Importantly, miR-26a could inhibit cancer cell growth in human liver cancer [15]. Our preliminary study using qRT-PCR analysis has found that miR-26a was sharply down-regulated in the regenerating liver tissues at 120 h following 70% PH compared with SH group in mice. In the present study, we further confirmed that miR-26a was obviously down-regulated during the proliferative phase of LR.

LR is a timely sequence of events consisting of priming phase, proliferation/expansion phase, and termination phase [37]. As expected, the LBWR increased gradually as the residual live mass increased, while the proliferation rate reached its peak at 36–72 h post-PH. Importantly, we found that miR-26a was down-regulated before 24 h post-PH, suggesting miR-26a may be a negative regulator of LR and took part in the regulation of LR at the very beginning. And we also demonstrated that over-expression of miR-26a could suppress the hepatocyte proliferation in LR. Together with the previous study [15], this study proved that miR-26a play important roles in inhibition of both hepatocyte proliferation during LR and cancer cell growth in liver carcinogenesis.

Undoubtedly, hepatocyte proliferation during LR requires active cell cycle progression. Cell cycle progression itself is regulated by cyclin expression and activation of cyclin-dependant kinases (CDKs) [37]. And the algorithms analysis predicts that miR-26a may regulate CCND2, CCNE1, CCNE2, and CDK6. It is well known that the D-type cyclins (D1, D2, and D3) play key roles in cell cycle machinery, and these cyclins positively regulate cell proliferation by binding to CDK4 and CDK6, resulting in the phosphorylation of the retinoblastoma protein and the G1/S transition of the cell [38]. As well, it is widely acknowledged that CCNE2 and CCNE1 are critically required for normal proliferation of virtually all mammalian cell types, especially in controlling transition of quiescent cells into cell cycle progression [39]. Herein, we showed that miR-26a over-expression inhibits expression of CCND2 and CCNE2, and conversely, anti-expression of miR-26a leads to an enhanced expression of CCND2 and CCNE2 in both mRNA and protein level *in vivo,* suggesting CCND2 and CCNE2 are probably the targeted genes of miR-26a. Together, miR-26a down-regulation probably regulates hepatocyte proliferation through enhancing CCND2 and CCNE2 expression. Understanding how miR-26a targets CCND2 and CCNE2 may provide detailed mechanism by which miR-26a regulates hepatocyte proliferation during LR.

There are a number of methods, such as placenta extract [40], platelet [41], and carbon monoxide [42], have been shown to promote LR in animal studies. However, none of these methods have been translated into clinical practice because of their low safety and efficacy. MiRNAs, as potent post-transcriptional regulators of gene expression, offer hopes of novel therapeutic targets for enhanced LR [43]. Of course, although the data are interesting, there are several limitations in this study. Firstly, the detailed molecular mechanism by which miR-26a regulates CCND2 and CCNE2 is still to be elucidated. In addition, how miR-26a may affect the two major LR pathway, namely IL-6 and hepatocyte growth factors (HGF) pathways, is unkown. These limitations provide room for future study.

In conclusion, we report for the first time that miR-26a plays crucial roles in regulating the proliferative phase of LR. These results show that miR-26a may regulate LR by repressing the expression of CCND2 and CCNE2. This study sheds lights on the mechanism by which miR-26a regulates LR during PH and may act as a therapeutic target in the future.

## Supporting Information

Figure S1 **Transfection reliability and** ***in vivo*** **bioluminescence imaging.** The area of bioluminescence was scarcely different among AA group (A), AM group (B) and AL group (C) at 24 h after transfection, suggesting that transfection efficiency among three groups was similar. The control group (no transfection) showed no bioluminescence image (D).
(TIF)

Figure S2 **CCND2 and CCNE2 are potential targeted genes of miR-26a in Nctc-1469 mouse liver cells.** (A) Anti-miR-26a expression increased the mRNA expression of CCND2 and CCNE2 as shown by qRT-PCR. Conversely, miR-26a over-expression declined the mRNA expression of these two genes. The mRNA expression of CCNE1 and CDK6 showed no obvious change. (B) Anti-miR-26a expression up-regulated the protein expression of CCND2 and CCNE2. In contrast, miR-26a over-expression down-regulated the protein expression of CCND2 and CCNE2. (C) The protein expression of CCNE1 and CDK6 showed no obvious change. ^*^ *P*<0.05, ^**^ *P*<0.01, ^***^ *P*<0.001.
(TIF)
