# Supplementary figures and images for: Down-Regulation of microRNA-26a Promotes Mouse Hepatocyte Proliferation during Liver Regeneration
Source: PLoS One. 2012 Apr 4;7(4):e33577. doi: 10.1371/journal.pone.0033577 (PMC3319545; doi:10.1371/journal.pone.0033577)

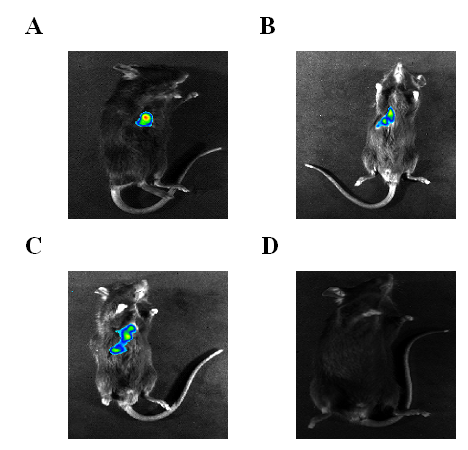

Supplement: Figure S1 — Transfection reliability and in vivo bioluminescence imaging. The area of bioluminescence was scarcely different among AA group (A), AM group (B) and AL group (C) at 24 h after transfection, suggesting that transfection efficiency among three groups was similar. The control group (no transfection) showed no bioluminescence image (D). (TIF) [file pone.0033577.s001.tif]

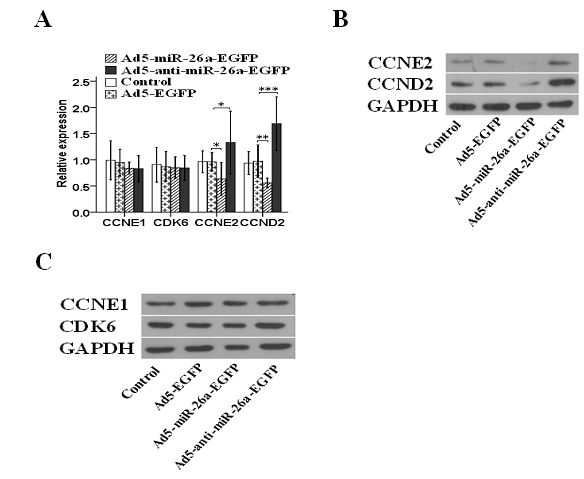

Supplement: Figure S2 — CCND2 and CCNE2 are potential targeted genes of miR-26a in Nctc-1469 mouse liver cells. (A) Anti-miR-26a expression increased the mRNA expression of CCND2 and CCNE2 as shown by qRT-PCR. Conversely, miR-26a over-expression declined the mRNA expression of these two genes. The mRNA expression of CCNE1 and CDK6 showed no obvious change. (B) Anti-miR-26a expression up-regulated the protein expression of CCND2 and CCNE2. In contrast, miR-26a over-expression down-regulated the protein expression of CCND2 and CCNE2. (C) The protein expression of CCNE1 and CDK6 showed no obvious change. * P<0.05, ** P<0.01, *** P<0.001. (TIF) [file pone.0033577.s002.tif]
